# Cognitive Impairment in the 3xTg-AD Mouse Model of Alzheimer’s Disease is Affected by Aβ-ImmunoTherapy and Cognitive Stimulation

**DOI:** 10.3390/pharmaceutics12100944

**Published:** 2020-10-02

**Authors:** Alejandro R. Roda, Gisela Esquerda-Canals, Joaquim Martí-Clúa, Sandra Villegas

**Affiliations:** 1 Protein Folding and Stability Group, Departament de Bioquímica i Biologia Molecular, Facultat de Biociències, Universitat Autònoma de Barcelona, 08193 Bellaterra, Barcelona, Spain; Alejandro.ramos@uab.cat (A.R.R.); Gisela.esquerda@uab.cat (G.E.-C.); 2 Departament de Biologia Cel·lular, de Fisiologia i d’Immunologia, Unidad de Citologia i d’Histologia, Facultat de Biociències, Universitat Autònoma de Barcelona, 08193 Bellaterra, Barcelona, Spain; Joaquim.marti.clua@uab.cat

**Keywords:** Aβ, tau, immunotherapy, Alzheimer, scFv, 3xTg-AD, training

## Abstract

Clinical symptoms of Alzheimer’s Disease (AD) include behavioral alterations and cognitive impairment. These functional phenotypes early occur in triple-transgenic (3xTg-AD) mice. Specifically, behavioral alterations are first detected when mice are at around 2.5 months old and cognitive impairment in between 3- and 5-month-old mice. In this work, the effect of chronic Aβ-immunotherapy on behavioral and cognitive abilities was tested by monthly administering the antibody fragment scFv-h3D6 to 3xTg-AD female mice from 5 to 9 months of age. An untreated group was used as a reference, as well as to attain some information on the effect of training during the longitudinal study. Behavioral and psychological symptoms of dementia (BPSD)-like symptoms were already evident in 5-month-old mice, in the form of neophobia and anxious-like behavior. The exploratory activity decreased over the longitudinal study, not only for 3xTgAD mice but also for the corresponding non-transgenic mice (NTg). Learning abilities of 3xTg-AD mice were not seriously compromised but an impairment in long-term spatial memory was evident at 5 months of age. Interestingly, scFv-h3D6-treatment affected the cognitive impairment displayed by 5-month-old 3xTg-AD mice. It is worth noting that training also reduced cognitive impairment of 3xTg-AD mice over the longitudinal study, suggesting that to properly quantify the isolated therapeutic potential of any drug on cognition using this model it is convenient to perform a prompt, age-matched study rather than a longitudinal study. In addition, a combination of both training and Aβ-immunotherapy could constitute a possible approach to treat Alzheimer’s disease.

## 1. Introduction

Alzheimer’s disease (AD) is the most prevalent cause of dementia worldwide [1]. Accumulation of the amyloid-β (Aβ) peptide in the neural tissue, either because of its overproduction or impaired clearance [2], is considered a key factor in the progression of this neurodegenerative disorder [3,4]. Furthermore, other age-related, protective or disease-promoting factors have been shown to be involved in the disease progression [5].

Clinical symptoms include both cognitive and behavioral alterations. The first consist mainly of memory loss and learning deficiencies, as well as impairments in other cognitive abilities interfering with mood, reason, judgment, and language [6,7]. The second comprises a wide range of neuropsychiatric symptoms clustered in psychotic (as delusion, hallucinations, and aberrant motor activity) and emotional symptoms (as agitation, dysphoria, anxiety, irritability, and apathy), and which are commonly referred to as behavioral and psychological symptoms of dementia (BPSD) [8,9,10].

The modeling of human AD in animals is being quite a challenging process. Neuropathological hallmarks were rather easy to reproduce in mice by introducing those mutations found in the familial form of the same, or related, diseases [11,12]. However, due to the heterogeneity of clinical symptoms [13] and the complexity of its evaluation [14,15,16], mimicking the cognitive and behavioral AD-phenotypes has become a rather difficult goal [17,18]. An overview of the phenotypes achieved in several mice models for AD is detailed by Webster et al. [19]. In brief, cognitive decline is generally detected early as impairments in spatial working memory [20], followed by disturbances in associative learning and reference memory [21,22], and eventually concluding with deficits in recognition memory [20,23,24]. Regarding emotional alterations, although delusion or hallucinations are human symptoms rather difficult to identify in mice, other disturbances such as neophobia, exploratory impairment, or anxiety have been characterized [25]. Despite the first conception about the late-onset of BPSD, these symptoms are currently demonstrated to already appear in the prodromal phases of AD [10].

The triple-transgenic mouse model of AD (3xTg-AD) reproduces human amyloid and tau pathologies through similar regional and temporal patterns [26,27]. In this model, cognitive impairment starts at young ages. Associative learning deficits are first detected at around 3–5 months of age, impairments in spatial working memory in the Morris water maze (MWM) paradigm at 6 months, deficits in recognition memory at around 9–11 months, and reference memory impairment in the Barnes maze task at 12 months [19]. In addition, behavioral alterations in 3xTg-AD mice occur at early ages. Alterations in exploratory activity are first detected in the open-field test (OFT) at 2.5 months of age (and at older ages in the corner test, CT), lower habituation to the novelty and uninhibited behavior at 6 months, and neophobia at 12 months, whereas increased emotionality is observed in most of the tests at the adulthood and elderly stages [25].

Taking into account the amyloid cascade hypothesis [28], enhancing Aβ clearance has become the main pharmacological approach tested for AD. Among different monoclonal antibodies (mAbs) that have been developed, bapineuzumab was the first one to reach phase III [29]. However, clinical trials were discontinued because of the occurrence of amyloid-related imaging abnormalities (ARIAs) and the absence of clinical benefit [30]. The latter has been attributed to the fact that the treatment started when the pathology was at an advanced stage. Because the occurrence of ARIAs is related to an immune over-activation via the Fc-receptor, the use of single-chain variable fragments (scFv), which lack the Fc fragment, has emerged as an alternative to immunization with full-length mAbs. In addition, we have learned from clinical trials that treatments for AD must be started at the prodromal phase, when the symptoms are not yet evident. The anti-Aβ scFv-h3D6, derived from bapineuzumab, has been effective in the prevention of Aβ-induced toxicity in SH-SY5Y neuroblastoma cell-cultures [31] and in ameliorating AD histologically hallmarks, without eliciting any detectable inflammatory response in young 3xTgAD mice [32,33,34,35]. Interestingly, a longitudinal study using magnetic resonance imaging and spectroscopy showed the efficacy of scFv-h3D6 treatment in preventing loss of brain volume occurring in 3xTg-AD mice [36]. However, cognitive improvement through a chronic treatment has not ever been assessed. Longitudinal studies are relevant for defining the progress of the disease, in both animal models and humans [37]. Additionally, the monitoring of biomarkers by biochemical analyses, image-based techniques, and/or functional and cognitive testing not only enables the further definition of the chronological evolution of the pathology but it is also crucial for early diagnosis and for evaluating therapies in both clinical and preclinical research [4,38].

In the present work, behavioral and cognitive impairments in 3xTg-AD female mice were longitudinally assessed, from 5 (young adulthood) to 9 (middle adulthood) months of age, and compared to the non-pathological ageing in the corresponding non-transgenic age and gender-matched mice (NTg, B6129SF2). In addition, the effects on cognitive functions after chronic administration of scFv-h3D6 to 3xTg-AD mice were tested. Interestingly, a positive effect of cognitive stimulation derived from the longitudinal training, which consisted in repeated MWM testing, was observed, not only in 3xTg-AD mice but also in NTg mice. Indeed, this result concurs with previous studies showing that periodic cognitive enrichment throughout aging improves memory performance in 3xTg-AD mice [39].

## 2. Material and Methods

### 2.1. ScFv-h3D6 Production

ScFv-h3D6 was recombinantly expressed in *Escherichia coli* Origami 2 (DE3)/pETtrx-1a and purified as previously described [31]. Briefly, after refolding by dilution, the Trx-tag was removed from the precursor construct by TEV (Tobacco Etch Virus) proteolysis followed by Immobilized Metal Affinity Chromatography (IMAC), and then, the scFv-h3D6 was purified by CEX cation-exchange chromatography ((CEX) Resource S column). Lipopolysaccharides were detached from the protein by using Detoxi-Gel Endotoxin Removing columns (ThermoFisher Scientific, Waltham, MA, USA).

### 2.2. Animals

The triple-transgenic mouse model of Alzheimer’s disease (3xTg-AD) was engineered at the University of California, Irvine, by introducing *APP_Swe_* and *MAPT_P301_*_L_ transgenes into a *PS1_M146V_* homozygous knock-in single-cell embryo [26]. Female mice were selected because they conserve the phenotype as originally described [40] and exhibit greater Aβ burden and larger behavioral deficits than age-matched male mice [41]. Animals used in the present work belong to the 3xTg-AD colony, and the corresponding non-transgenic (NTg) mice (B6129SF2), stablished by our group in the Animal Facility (Servei d’Estabulari) at the Universitat Autònoma de Barcelona (UAB). Founder animals were provided by The Jackson Laboratory, Bar Harbor, ME, USA. Animals were maintained under standard laboratory conditions (temperature of 22 ± 2 °C and relative humidity of 55 ± 5%, a 12 h light:dark cycle starting at 08:00 a.m., food and water provided ad libitum).

All the experiments were approved by the UAB Animal Research Committee and the Government of Catalonia (CEEAH 0661) and performed in accordance with the Guide for the Care and Use of Laboratory Animals published by the US National Institutes of Health.

### 2.3. Experimental Design

**Figure 1:**
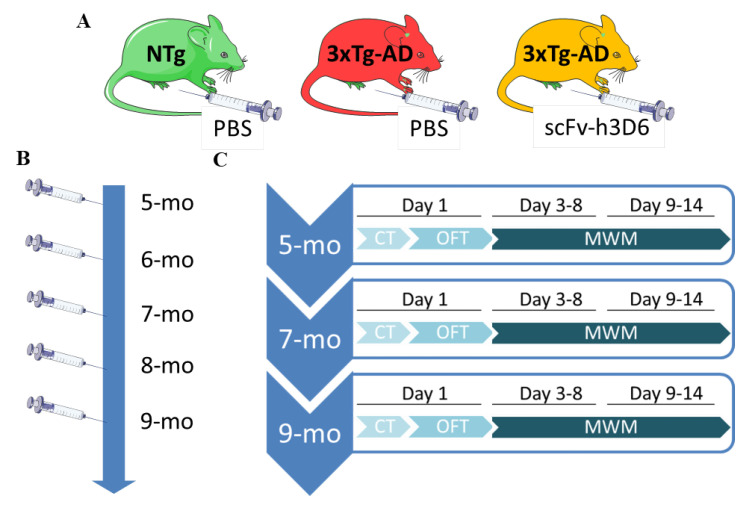
Experimental Design. (**A**) Mice were divided into 3 experimental groups (*n* = 10 each group): PBS-treated Non-Transgenic mice (NTg); PBS-treated 3xTg-AD mice, and scFv-h3D6 treated 3xTg-AD mice. (**B**) Once a month, starting at 5 months of age, mice were intraperitoneally administered with the treatment (100 μg of scFv h3D6) or with the vehicle (PBS). (**C**) Behavioral and cognitive testing was longitudinally assessed at 5, 7, and 9 months of age 24 h after the corresponding administration. The sorting of the tests was performed based on the degree of stress in each test, with the most stressful one at the end. The corner test (CT) and open-field test (OFT) were carried out on day 1 and the Morris water maze (MWM) on days 3–14 (3–8 MWM, 9–14 reversal MWM).

The experimental design is schematized in Figure 1. Twenty 5-month-old 3xTg-AD female mice and 10 gender- and age-matched NTg mice were used in this study. The 3xTg-AD mice were randomly distributed into 2 groups, PBS-treated 3xTg-AD mice and scFv-h3D6-treated 3xTg-AD mice (*n* = 10 each group). NTg mice (*n* = 10) were PBS-treated to obtain references for non-pathological condition and natural aging. Once a month, starting at 5 months of age, mice were intraperitoneally administered with 100 µg of scFv-h3D6 diluted in 200 µL of PBS or with the vehicle (PBS). Behavioral and cognitive testing was performed at 5, 7, and 9 months of age and started 24 h after the corresponding administration (see Figure 1). Two PBS-treated 3xTg-AD mice died at 8 months of age and, therefore, *n* = 8 was used for this group at 9 months of age. All the experiments and analyses were carried out in a blind fashion.

Statistical analyses were performed using Graphpad Prism v6 software. Results are expressed as means ± SD. To check sample normality, the D’Agostino Pearson omnibus K2 test was performed. The effects of the factors group (NTg, PBS-treated 3xTg-AD or scFv-h3D6-treated 3xTg-AD) and time (*t*) were analyzed with repeated measures (for time factor) two-way ANOVA. Tukey’s multiple comparisons test was performed to compare groups between one another. To evaluate differences among all groups in immunohistochemical analysis, non-repeated measures one-way ANOVA with Tukey’s multiple comparisons test was used. A *p*-value ≤ 0.05 was considered statistically significant. A *p*-value over 0.05 and minor or equal to 0.1 (0.05 < *p* ≤ 0.1) was considered as a low, but not significant value. These low values were considered since the real relevance of *p* values is being questioned by several researchers [42].

### 2.4. Sample Collection and Processing

Animals were anesthetized using 1% isoflurane (Esteve) (Barcelona, Spain) and euthanized by decapitation [43]. Brains were immediately removed from the skulls, rinsed in cold PBS, weighed, and dissected on ice. Brains were rapidly fixed by immersion in 4% paraformaldehyde (PFA) diluted in PBS for 48 h. Samples were embedded in paraffin following common procedures, serially sectioned in the coronal plane (10-µm thick), and mounted on SuperfrostTM Plus microscope slides (Thermo Fisher Scientific).

### 2.5. Immunohistochemistry

Sections were deparaffinized by immersion in xylene and rehydrated in serial dilutions of alcohol (*n* = 8, each group, to avoid unbalanced groups). Endogenous peroxidase activity was blocked by immersion in 3% H_2_O_2_ in pure methanol for 10 min. Antigen retrieval was achieved by immersion in 0.01M citrate buffer (pH 6.0) supplemented with 0.1% Tween-20 (Sigma-Aldrich Química SL, Saint Louis, MO, USA) at 96 °C for 20 min. Then, tissues were cooled at room temperature (RT) in PBS-T (PBS (pH 7.6), 0.1% Tween-20) and incubated in blocking buffer (5% normal goat serum (NGS, Sigma-Aldrich Química SL) and 5% bovine serum albumin (BSA, Sigma-Aldrich Química SL) in PBS-T to avoid non-specific binding. Slides were incubated overnight at 4 °C with the corresponding primary antibody (mouse anti-Aβ 6E10 mAb, 1:200, Covance Signet, ref. SIG-39320-200, lot. D13BF00601; or mouse monoclonal anti-human tau mAb, HT7, 1:100 Thermo Scientific, ref. MN1000, lot. TA2494341). After incubation with the secondary antibody and extravidin (Mouse ExtrAvidin Peroxidase Staining Kit antibody, Sigma-Aldrich Química SL, ref. EXTRA2-1KT), the slides were visualized using 3-3′ Diaminobenzidine (DAB, Sigma-Aldrich Química SL).

### 2.6. Image Capture and Processing

Bright-field images were captured using a Leica DMRB microscope (Leica, Wetzlar, Germany,) and a Leica DFC 500 camera (Leica) with a Leica PL Fluotar lens. The cerebral sections corresponded to the range of coordinates from Figure 43 (interarual 2.34 mm and Bregma −1.46 mm) to Figure 48 (interaural 1.74 mm and Bregma −2.06 mm) of The Mouse Brain in Stereotaxic Coordinates [44]. To quantify the percentage of immunoreactive 6E10 (Aβ) and HT7 (total tau protein) area, 2–3 sections of the hippocampus were used. This was so because previous studies indicate that scFvs penetrates and accumulates mainly in the periventricular areas, such as the hippocampus [45,46,47]. Moreover, intraneuronal Aβ and tau pathologies in the hippocampus of 3xTg-AD mice have been directly related with impaired cognitive abilities [48,49]. The percentage of immunoreactive area was calculated from binary images using the ImageJ software 1.8.0 (NIH, Bethesda, USA).

### 2.7. Behavioral and Cognitive Tests

Behavioral and cognitive tests started 24 h after treatment administration at 5, 7, and 9 months of age (Figure 1). The temporal sequence of behavioral testing was performed based on the degree of stress in each test, with the most stressful one at the end: the CT and OFT first, and the MWM on days 3–14. All the tests were recorded by a digital USB camera (The Imaging Source, Bremen, Germany) and processed with the ANY-maze software (v. 5.14, Stoelting Europe, Dublin, Ireland). Rearings, self-groomings, and defecations in the CT and OFT were manually quantified. All the material involved in the behavioral testing was thoroughly cleaned with 20% ethanol between trials to ensure the absence of olfactory cues.

*Corner test*. The CT was performed by placing the mice in the middle of a standard home-cage (makrolon, 22 × 22 × 14.5 cm) for 30 s. Horizontal activity (distance traveled, number of visits to the corner, latency of the first corner visit, and time spent in the corner), vertical activity (number of rearings and latency of the first rearing), and other emotional behaviors (immobility time and number of defecations) were recorded for evaluation.

*Open-field test*. The OFT consisted of an arena (white polyethylene, 42 × 42 × 50 cm) where the animals were placed for 15 min [50]. Horizontal activity (total distance traveled, entries to a virtually delimited central area, time spent and distance traveled in the central area and in the periphery, and ratios of central/peripheral time and distance), vertical activity (number of rearings and latency of the first rearing) and other emotional behaviors (number of self-groomings, latency of the first self-grooming, and total time on self-grooming) were recorded for evaluation.

*Morris water maze test*. The Morris water maze (MWM) paradigm assesses the hippocampal-dependent learning and memory by testing the ability to find a hidden platform in a pool full of opaque water stained with non-toxic white tempera (Ø, 120 cm for the pool, Ø, 11 cm for the platform) [51,52]. The paradigm comprised 3 phases: (1) *Visible platform*. The first day consisted in the visible platform stage, in which the platform was highlighted by a colored flag. Animals performed 4 trials, with an inter-trial period of 10 min, to ensure that they realized of the existence of the platform. (2) *Acquisition phase*. During the acquisition phase, the platform was hidden ~5 mm under the water surface and cues were added to make spatial orientation possible. This phase consisted of 4 trials per day, for 5 consecutive days, in which the animals should reach the platform upon being oriented by the cues. Mice were manually guided to the platform when they were unable to find it in 60s. Starting points were randomly ordered to ensure different sequences among stages and days. Since each trial was started from a different virtual cardinal point, values obtained from each trial were normalized by the corresponding starting point-platform distances. (3) *Probe*. The platform was removed for the final probe. This phase consisted of 60s of navigation in absence of the platform and was performed 24 h after the last acquisition stage to properly evaluate long-term memory. Animals started from the most distant point to the usual platform location. The reversal test consisted of the acquisition and the probe phases but replacing the platform in a different location.

Once removed from the water, animals were located under a heating lamp (red-light, 100 W) to prevent hypothermia. Mean swimming speed and distance traveled were measured across trials during the acquisition stages. Latency to the platform, platform crossings and path efficiency, as well as time and distance traveled within a virtual zone centered on the former platform location (normalized by areas), were measured in the final probe.

## 3. Results

### 3.1. Progression of BPSD-Like Symptoms

**Figure 2:**
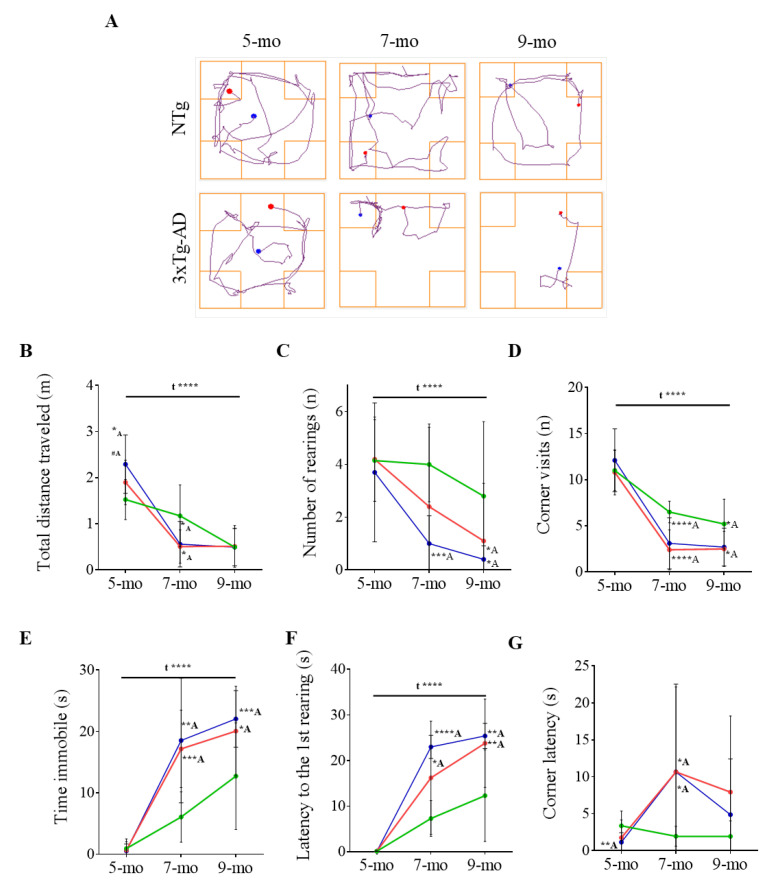
Behavioral and psychological symptoms of dementia (BPSD)-like symptoms as tested in the corner test. (**A**) Track plots of the pathway followed by PBS-treated NTg and 3xTg-AD representative animals. The blue point indicates the animal location at the starting point and the red point the final location. (**B**–**F**) Evolution of NTg (green), 3xTg-AD (red), and scFv-h3D6-treated 3xTg-AD (blue) mice with age considering (**B**) the distance travelled (m), (**C**) number of rearings, (**D**) number of corner visits, (**E**) immobility time (s), (**F**) latency to the first rearing (s), and (**G**) latency to the first corner visit (s). Values are represented by means and error bands correspond to SD. # indicates a low, but not significant, *p*-value (0.05 < *p* ≤ 0.1) (Total distance travelled (m), 5-mo, NTg vs. 3xTg-AD-PBS, *t* = 2.022, *p* = 0.0583* significant differences *p* ≤ 0.05, ** *p* ≤ 0.01, *** *p* ≤ 0.001, **** *p* ≤ 0.0001. ^A^ refers to NTg mice.

**Figure 3:**
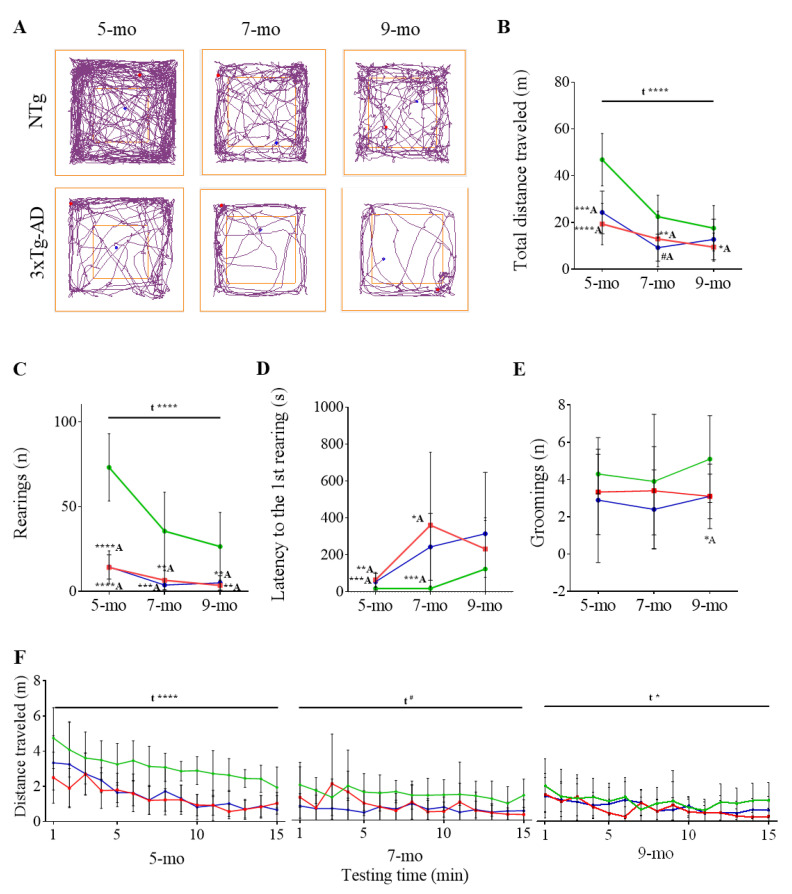
BPSD-like symptoms as tested in the open-field test. (**A**) Track plots of the pathway followed by PBS-treated NTg and 3xTg-AD representative animals. The blue point indicates the animal location at the starting point and the red point the final location. (**B**–**E**) Evolution of NTg (green), 3xTg-AD (red), and scFv-h3D6-treated 3xTg-AD (blue) mice with age considering B) the distance travelled (m), (**C**) number of rearings, (**D**) latency to the first rearing (s), and (**E**) number of rearings. (**F**) Distance travelled over-time at different ages. Values are represented by means and error bands correspond to SD. # indicates a low, but not significant, *p*-value (0.05 < *p* ≤ 0.1) (Total distance travelled (m), 7-mo, NTg vs. 3xTg-AD-PBS, *t* = 2.022, *p* = 0.0583). * significant differences *p* ≤ 0.05, ** *p* ≤ 0.01, *** *p* ≤ 0.001, **** *p* ≤ 0.0001. ^A^ refers to NTg mice.

BPSD-like symptoms were evaluated by the corner test and the open-field test (Figure 2 and Figure 3 and Table 1 and Table 2). Both PBS-treated NTg and 3xTg-AD mice exhibited similar performances at 5 months of age in most of the parameters analyzed in the CT (Figure 2, Table 1). However, track plots of the pathway followed by animals showed dramatic differences upon aging (Figure 2A). In both groups, the distance traveled shortened over time (F _(2, 52)_ = 32.32, *p* ≤ 0.0001) (Figure 2B) concomitantly with a decrease in the number of rearings (F _(2, 51)_ = 7.507, *p* = 0.0014) (Figure 2C) and in the number of corner visits (F _(2, 52)_ = 63.95, *p* ≤ 0.0001) (Figure 2D), and an increase in the immobility time (F _(2, 52)_ = 39.36, *p* ≤ 0.0001) (Figure 2E) and in the latency to the first rearing (F _(2, 51)_ = 35.25, *p* ≤ 0.0001) (Figure 2F). Altogether this evidences a reduction in the exploratory activity upon aging in both genotypes. However, 3xTg-AD mice exhibited a more pronounced inactive behavior indicating a higher vulnerability to the novelty. This is especially evident since mice are 7-month-old. At 9 months of age and compared to NTg mice, 3xTg-AD mice showed increased immobility time (*t* = 1.984, *p* = 0.0064) and latency to the first rearing (*t* = 3.147, *p* = 0.0062) and decreased number of rearings (*t* = 2.354, *p* = 0.0317) and number of corner visits (*t* = 2.296, *p* = 0.0356). These data showed an enhanced anxious-like behavior in the 3xTg-AD female mice that exacerbated over-time.

In the OFT (Figure 3, Table 2), 5-month-old 3xTg-AD mice traveled a shorter distance (*t* = 6.107, *p* ≤ 0.0001) (Figure 3A,B), displayed a lower number of rearings (*t* = 7.922, *p* ≤ 0.0001) (Figure 3C), and an increased latency to the first rearing (*t* = 3.336, *p* = 0.0030) (Figure 3D) than the NTg mice. This agrees with the reduced exploratory activity observed in the CT. In consonance, other traits such as the lower number of self-groomings (Figure 3E), even though statistical significance was not reached, and the larger latency to the first one (Table 2) (*t* = 2.240, *p* = 0.0388) pointed to the inactivity of the 3xTg-AD mice already at 5 months of age. The representation of the distance travelled during the test shows an evident decrease over time indicative of a habituation process in both groups (F _(14, 257)_ = 7.897, *p* ≤ 0.0001) (Figure 3F).

Similarly to what was observed in the corner test, the exploratory activity was reduced over time. This behavior is shown by the decrease in the total distance travelled (F _(2, 54)_ = 22.96, *p* ≤ 0.0001) and in the number of rearings (F _(2, 52)_ = 17.73, *p* ≤ 0.0001) and the increase in the latency to the first rearing (F _(2, 52)_ = 3.016, *p* = 0.0576). Nevertheless, 9-month-old 3xTg-AD female mice exhibited a much larger decrease in exploratory activity than NTg mice (Figure 3).

In summary, BPSD-like symptoms were already evident in 5-month-old 3xTg-AD mice and exacerbated upon aging. Such an early onset and exacerbation of BPSD-like symptoms are triggered by the disease, but these exacerbated symptoms also occur late in the NTg group because of natural aging.

### 3.2. Effect of Cognitive Stimulation in Spatial Learning and Memory

**Figure 4:**
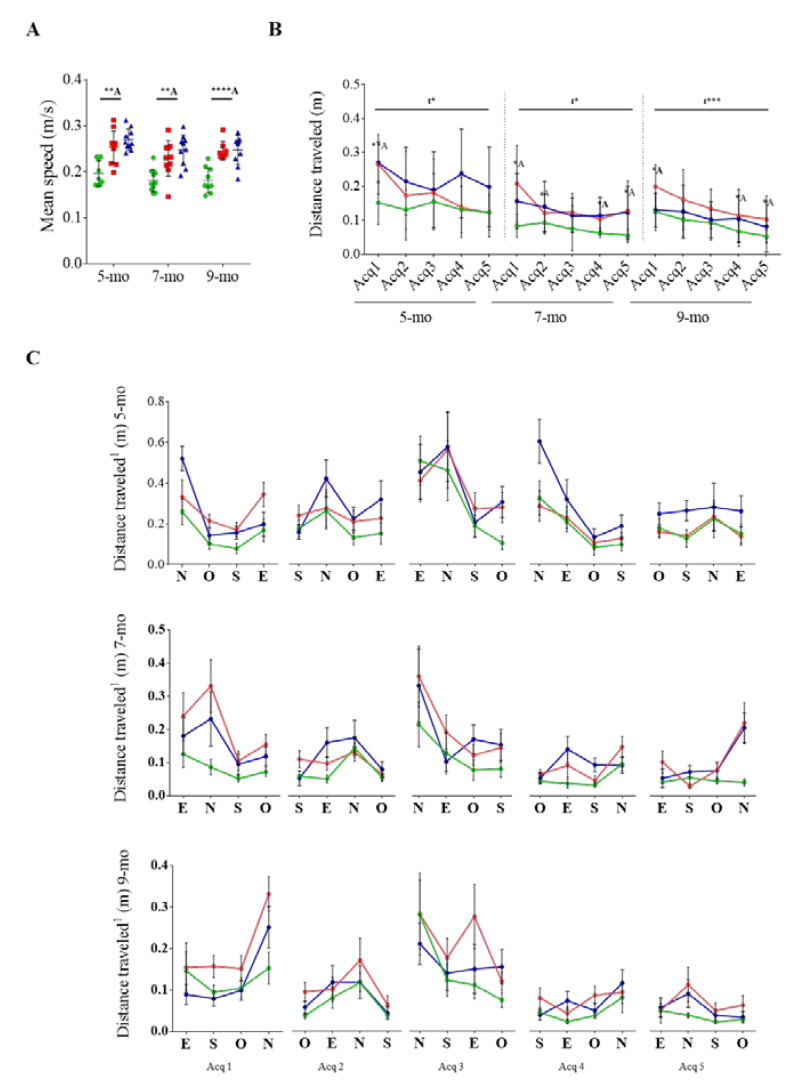
Longitudinally assessed acquisition phase in the MWM paradigm. NTg mice in green, 3xTg-AD mice in red, and scFv-h3D6-treated 3xTg-AD in blue. (**A**) Averaged swimming speed. (**B**) Distance traveled in each acquisition stage. (**C**) Detailed distances traveled in each trial, from the preliminary visible-platform phase to the last acquisition stage. Values are represented by the mean and error bands correspond to SD. # indicates a low, but not significant, *p*-value 0.05 < *p* ≤ 0.1 (Distance travelled (m), 9-month, Acq 2, NTg vs. 3xTg-AD-PBS, *t* = 1.770, *p* = 0.0957), * significant differences *p* ≤ 0.05, ** *p* ≤ 0.01, *** *p* ≤ 0.001, **** *p* ≤ 0.0001. ^A^ refers to NTg mice.

Spatial learning and memory were evaluated by the MWM paradigm (Figure 4). 3xTg-AD mice exhibited a higher swimming speed than NTg mice at all studied ages (*t* = 3.870 *p* = 0.0012; *t* = 3.347 *p* = 0.0036; *t* = 5.710 *p* ≤ 0.0001, respectively) (Figure 4A), supporting the anxious-like behavior observed in the CT and OFT.

Due to the differences in the speed, the efficiency to reach the platform was measured by the distance traveled to the platform instead of the platform latency (Figure 4B). First, motor or visual impairments were discarded in the visible-platform paradigm because all mice reached the platform in less than 60 s. Then, spatial learning was assessed during the acquisition stages of the MWM paradigm (Figure 4C). Five-month-old 3xTg-AD mice traveled longer distances than age-matched NTg mice at the first acquisition day (*t* = 3.179, *p* = 0.0055). However, the pathway was progressively improved (F _(4, 85)_ = 2,922, *p* = 0.0257) and reached values similar to NTg group at the end of the spatial acquisition test, which indicated that there was no learning impairment. This profile was also shown at 7 and 9 months of age but the values of the NTg group at the last acquisition day were not reached (*t* = 2.699, *p* = 0.0147; *t* = 2.246, *p* = 0.0392) (Figure 4B). In contrast, NTg mice straightforwardly reached the platform at all the studied ages.

**Figure 5:**
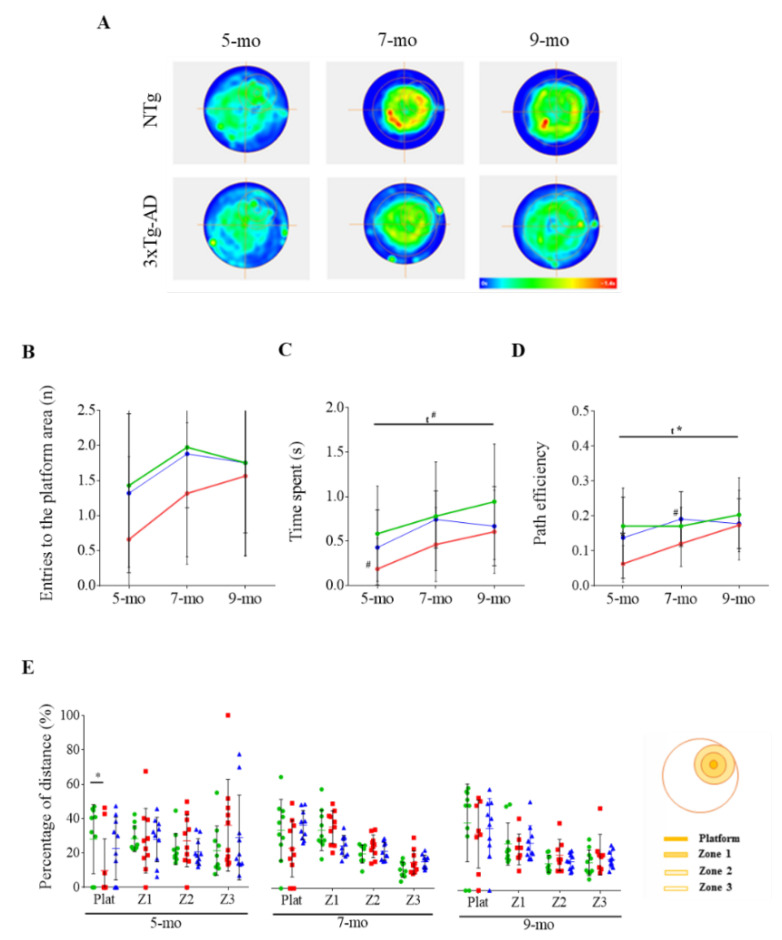
Long-term memory as assessed by the probe trial in the MWM. (**A**) Mean heat maps of the 5-, 7- and 9-month-old 3xTg-AD and NTg mice locations. Scale from blue (unoccupied regions) to red (the most occupied regions). (**B**–**D**) Evolution of NTg (in green), 3xTg-AD (in red), and scFv-h3D6-treated 3xTg-AD (in blue) mice with age considering (B) the number of entries (n), (**C**) time spent in the platform zone (s), and (**D**) path efficiency to the platform zone. (**E**) Comparative percentage of the distance traveled in each region (platform area, first concentric region (Z1), intermediate region (Z2), and external region (Z3)) between NTg and 3xTg-AD mice. Values are represented by means and error bands correspond to SD. # indicates a low, but not significant, *p*-value 0.05 < *p* ≤ 0.1 (Time spent in the platform zone (s), 5-mo, NTg vs. 3xTg-AD-PBS, (*t* = 1.965, *p* = 0.0651; Path efficiency, 7-mo, NTg vs. 3xTg-AD-PBS, *t* = 1.871, *p* = 0.0778). * significant differences *p* ≤ 0.05. ^A^ refers to NTg mice.

Twenty-four hours after the acquisition phase, long-term memory was assessed in the probe trial (Figure 5). At 5 months of age, NTg mice tend to present higher values than 3xTg-AD mice for entries to the platform area (Figure 5B) and time spent within the region (Figure 5C) (*t* = 1.965, *p* = 0.0651), as well as a better pathway efficiency (Figure 5D) (*t* = 2.827, *p* = 0.0112). NTg mice also swam a higher distance across the platform area than 3xTg-AD mice (*t* = 2.117, *p* = 0.0485), and performed an overall platform-directed ordered pattern in contrast to the random pattern performed by 3xTg-AD mice (Figure 5E). A progressive improvement in the performance was observed over time in the probe test (Figure 5). Both groups tended to increase the number of entries to the platform area and the time spent in the platform zone (F _(2, 52)_ = 2.733, *p* = 0.0743). The pathway efficiency was improved over time (F _(2, 52)_ = 3.435, *p* = 0.0347). Moreover, differences that were evident in the distance travelled in the platform zone at 5 months of age later disappeared. At 9 months of age, differences between NTg and 3xTg-AD mice were not detected in the probe test, even though NTg mice seemed to display a slightly improved performance.

As a conclusion, there was no learning impairment in the 3xTg-AD mice at 5 months of age, but long-term memory was affected. In addition, and although both genotypes improved after training, the 3xTg-AD mice exhibited greater improvements, which shows that cognitive stimulation is beneficial to prevent cognitive impairment even in pathological conditions.

### 3.3. Reversal Learning and Memory and Their Evolution

**Figure 6:**
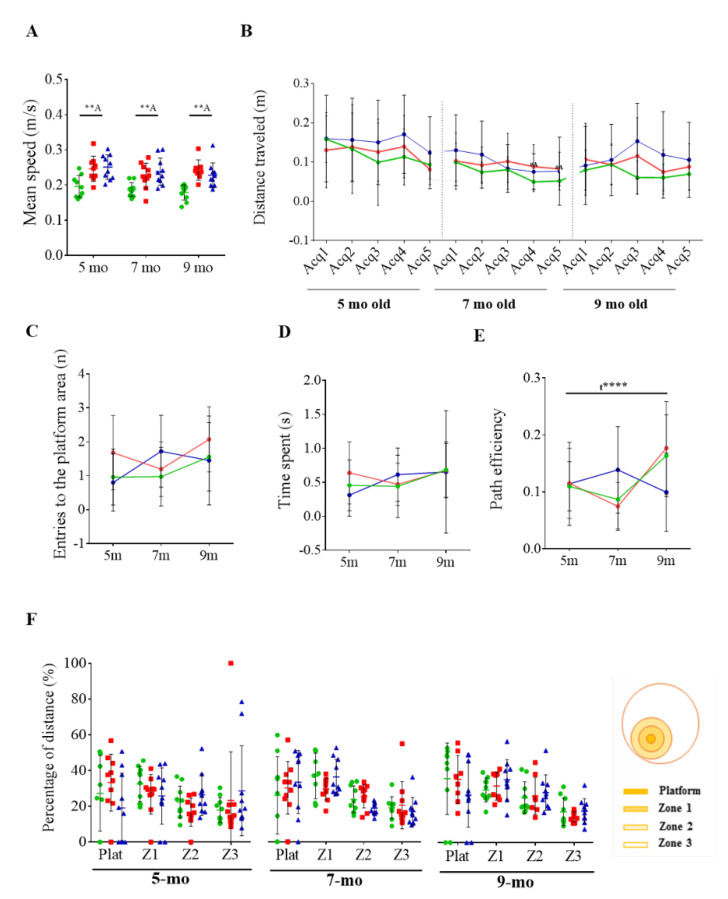
Reversal testing in the MWM. (**A**) Averaged swimming speed of non-transgenic (NTg, in green), triple-transgenic (3xTg-AD, in red) mice, and scFv-h3D6-treated 3xTg-AD (in blue). (**B**) Distance traveled in each acquisition stage (averaged from four trials). (**C**–**E**) Evolution over ages by considering (**C**) the number of entries (n), (**D**) the time spent in the platform zone (s), and (**E**) the path efficiency to the platform zone. (**F**) Percentage of distance traveled in each region (platform area, first concentric region (Z1), intermediate region (Z2), and external region (Z3)). Values are represented by means and error bands correspond to SD. # indicates a low, but not significant, *p*-value 0.05 < *p* ≤ 0.1 (Distance travelled (m), 7-month, Acq 4, NTg vs. 3xTg-AD-PBS, *t* = 2.001, *p* = 0.0607; Distance travelled (m), 7-month, Acq 5, NTg vs. 3xTg-AD-PBS, *t* = 1.994, *p* = 0.0615). * significant differences *p* ≤ 0.05, ** *p* ≤ 0.01 ^A^ refers to NTg mice.

Once learning and memory abilities were analyzed, the extinction of initial learning and ability of acquiring a direct path to the new goal position were evaluated by the reversal test. The swimming speed of 3xTg-AD mice was still higher than that of NTg mice in all the ages tested (*t* = 3.128, *p* = 0.0065 at 5 months of age; *t* = 3.018, *p* = 0.0074 at 7 months; *t* = 5.4348, *p* ≤ 0.0001 at 9 months) (Figure 6A). At 5 months of age, both genotypes seemed to show the ability of learning where the new platform was located in the reversal acquisition test (Figure 6B). However, statistical significance was not achieved. In this sense, it should be considered that mice do not completely abandon their initial learning strategy [52]. Thus, it is rather difficult to evaluate whether there were differences or not between genotypes. At 7 and 9 months of age, 3xTg-AD mice showed a more random pattern during the acquisition, which could be related with difficulties for extinguishing previous spatial learning. This was also the case for 9-month-old NTg mice.

Related to the difficulties observed in both groups in properly extinguishing previous spatial learning during the reversal acquisition, non-differences in memory in the reversal probe were detected at any analyzed age (Figure 6C–F). In contrast with the progressive improvement observed in the probe test, only a positive effect was detected in the path efficiency over time (F _(2, 52)_ = 10.88, *p* ≤ 0.0001) during the reversal probe test.

Considering that even NTg mice showed troubles for extinguish spatial learning, differences between genotypes and the effect of the training are difficult to be evaluated with a spatial reversal paradigm.

### 3.4. Effects of Chronic Immunotherapy in 3xTg-AD Mice

Unfortunately, BPSD-like symptoms were not ameliorated after scFv-h3D6 treatment (Table 1 and Table 2, Figure 2 and Figure 3). However, spatial memory seemed to be partially recovered after treatment already in 5-month-old 3xTg-AD mice (Figure 5). In the probe test of the MWM paradigm, PBS-treated 3xTg-AD mice showed a reduction in the number of entries and in time spent in the platform area and a reduced path efficiency, as mentioned above. After scFv-h3D6 administration, differences between NTg and 3xTg-AD were not detected at 5 months of age. The number of entries (Figure 5B), the time spent in the platform area (Figure 5C), and the path efficiency (Figure 5D) were decreased in PBS-treated mice, but no differences between NTg and 3xTg-AD mice after scFv-h3D6 the treatment were found (*t* = 0.2137, *p* = 0.8332; *t* = 0.7130, *p* = 0.4850; *t* = 0.9540, *p* = 0.3527, respectively). The random pattern performed by 3xTg-AD mice also disappeared after treatment (Figure 5E). Moreover, no differences in distance travelled in the platform zone were detected between NTg and treated 3xTg-AD mice (*t* = 0.6370, *p* = 0.5322).

As PBS-treated 3xTg-AD mice improved their spatial memory over time, the effect of scFv-h3D6 treatment was not observed at 7 and 9 months of age. However, it is likely that scFv-hD6 was effective at 5 months of age. Therefore, cognitive stimulation as a consequence of training in a longitudinal study can mask the effect of an effective drug and the testing should rather be performed promptly and be accompanied by molecular probes.

### 3.5. 3xTg-AD Mice Exhibit Evident Amyloid and Tau Pathologies at 9 Months of Age

The 3xTg-AD mouse model is characterized by the development of both histological hallmarks of AD, senile plaques composed of Aβ and neurofibrillary tangles (NFT) of tau protein [26]. To confirm that these features were replicated in mice in this study, immunohistochemistry of NTg and 3xTg-AD mice for both, amyloid and tau pathologies, was performed.

**Figure 7:**
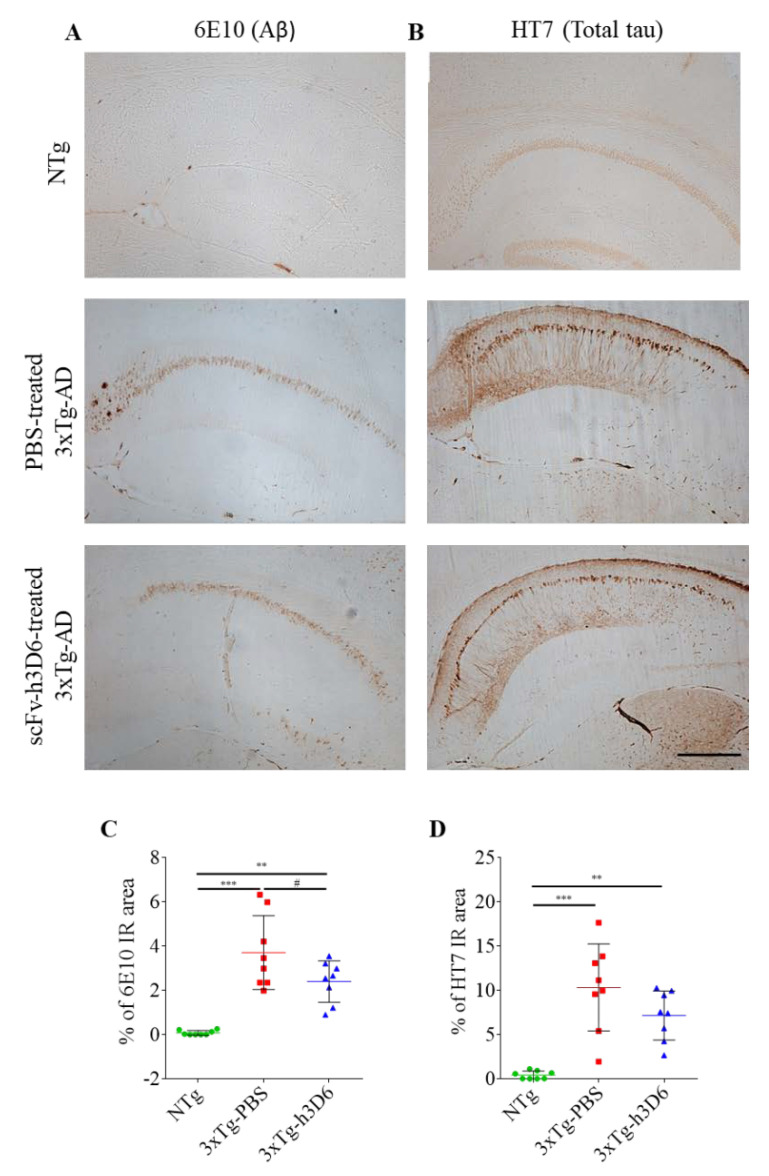
Aβ and tau deposition in 9-month-old 3xTg-AD hippocampus. (**A**) 6E10 (Aβ) and (**B**) HT7 (total tau) staining in 9-month-old NTg, PBS-treated 3xTg-AD and scFv-h3D6-treated 3xTg-AD mice. Scale bar 200 µm. (**C**) Percentage of 6E10 immunoreactive area in the hippocampus. (**D**) Percentage of HT7 immunoreactive area in the hippocampus. Values are represented by means and error bands correspond to SD. # indicates a low, but not significant, *p*-value (0.05 < *p* ≤ 0.1) (% of 6E10-immunoreactive area, 3xTg-AD-PBS vs. 3xTg-AD-scFv-h3D6, *t* = 1.932, *p* = 0.0738). ** *p* ≤ 0.01, *** *p* ≤ 0.001.

At 9 months of age, diffuse amyloid plaques in the subiculum of 3xTg-AD and intraneuronal Aβ pathology, mainly in the CA1 of the hippocampus, could be clearly seen (Figure 7A). As a reference, only intraneuronal Aβ pathology in the hippocampus was reported at 5 months of age [53]. In addition, total tau staining was also evident in 9-month-old 3xTg-AD mice. It is worth noting that spatial pattern of tau deposition in the hippocampus of 3xTg-AD mice was similar to Aβ distribution and was specially accumulated in the CA1 (Figure 7B). This is in consonance with the extensively reported downstream relation between both, amyloid and tau pathologies [54]. Thus, both amyloid and tau pathologies are properly developed in 3xTg-AD mice used in this study.

Interestingly, even though Aβ and tau pathologies are still evident in scFv-h3D6-treated 3xTg-AD mice, it could be appreciated that 6E10 immunoreactivity, particularly in the subiculum, is weaker than in PBS-treated 3xTg-AD mice. Indeed, the 6E10 immunoreactive area tended to be reduced in scFv-h3D6-treated mice (*t* = 1.932, *p* = 0.0738) (Figure 7C). This is in consonance with the extensively reported ability of scFv-h3D6 to reduce Aβ burden in the 3xTg-AD mouse model [32,33,34,35,43,55], which is behind the improvement in cognitive functions after treatment. Regarding tau pathology, even though significance was not achieved, a slight reduction in HT7 immunoreactive area could qualitatively be observed (Figure 7D). We previously demonstrated that scFv-h3D6 administration reduces total tau levels in 22-month-old 3xTg-AD females [47]. However, it is worth noting that the doses administrated in current study was smaller than in the study at 22 months of age, and so the effect is not so evident.

## 4. Discussion

In spite of the cause of AD remaining unknown, it is known that familial AD (FAD) is due to mutation in the amyloid precursor protein (APP) or in presenilins 1 or 2 (PSEN 1/2). However, only some risk factors, such as carrying the *apoEε4*-allele, have been related with the far more prevalent sporadic form of the disease (SAD) (at around 99% of AD cases) [56]. Thus, it is not fully understood why Aβ accumulation occurs and how it triggers tau hyperphosphorylation and eventually neural death leading to cognitive impairment and behavioral and psychological symptoms of dementia. Nevertheless, several risk factors like diabetes, obesity, a sedentary life style, among others, has been reported in the last few years [57].

**Figure 8:**
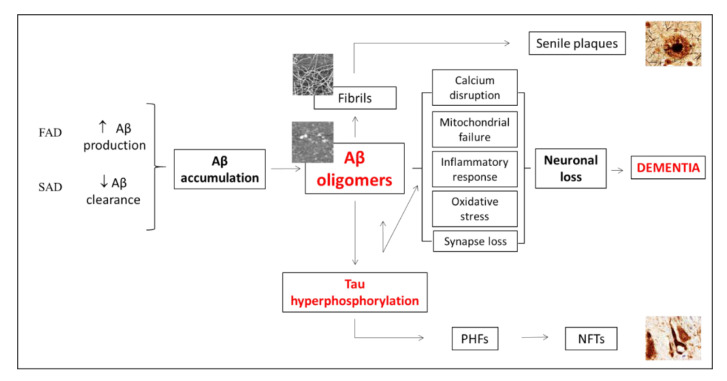
Putative mechanism of the effect of Aβ immunotherapy on cognition. The amyloid cascade hypothesis states that the accumulation of Aβ oligomers is toxic for neurons through two mechanisms, directly and by inducing tau hyperphosphorylation and aggregation. Tau aggregates, in turn, induce Aβ oligomerization, which leads to a positive feedback that potentiates the amyloid pathway and eventually results in neuronal loss and dementia. Thus, capturing Aβ oligomers prevents neuronal loss and the subsequent dementia. FAD: Familial AD. SAD; Sporadic AD. PHF: Paired helical filaments. NTFs: neurofibrillary tangles.

Interestingly, both Aβ and tau accumulation initially appear in the caudal hippocampus of 3xTg-AD mice [58]. Intraneuronal Aβ deposition, which appears early in the subiculum and hippocampal CA1 region, has been directly related with the first cognitive impairments detected in this mouse model [48,59]. We also observed here that the subiculum and hippocampal CA1 region are particularly affected by both amyloid and tau pathologies. Why these regions are more vulnerable needs to be elucidated. However, magnetic resonance imaging (MRI) studies showed that subiculum and CA1 atrophies are significantly increased in AD patients [60,61]. Thus, it seems that the rostral hippocampus is, at least in the early stages, more resilient to develop AD-like pathology and that the subiculum and hippocampal CA1 are the regions involved in the first cognitive impairment detected. Therefore, reducing early amyloid and tau accumulations in the caudal hippocampus could prevent cognitive impairment associated to AD progression (Figure 8).

Positive effects of Aβ immunotherapy in mice models have been reported [31,32,33,35], but, unfortunately, human clinical trials have not yet been successful in passing phase 3 [30]. There are also studies that suggest that physical exercise decreases synaptic dysfunction in AD mice models [62] and the positive effect of cognitive stimulation to delay the progression of the pathology [63].

This work details the neuropsychiatric-like symptoms and the cognitive impairment of the 3xTg-AD model from 5 to 9 months of age, and the effect of Aβ immunotherapy and cognitive stimulation in such a longitudinal study.

In 5-month-old 3xTg-AD mice, BPSD-like symptoms were evident in the OFT when comparing with the NTg mice. Thus, a reduction in the distance travelled and number of rearings, as well as an increased latency to the first rearing, could be quantified. Although differences were not detected in the CT at 5 months of age, neophobia was evident at 7 and 9 months in the 3xTg-AD mice. Specifically, the distance travelled, corner visits, and number of rearings were notably reduced and immobility time and latency to the first rearing increased. In general, a decreased in activity over-time could be observed not only in 3xTg-AD but also in NTg mice, probably because of normal aging. However, 3xTg-AD mice always exhibited less activity than NTg mice. Thus, it is likely that the pathology triggers early exacerbated BPSD-like symptoms that appear later in NTg mice due to normal aging. Unfortunately, scFv-h3D6 was not able to ameliorate BPSD-like symptoms detected in the 3xTg-AD, maybe as a consequence of the high amyloid burden observed in the amygdala already at 5 months old, as previously reported [53]. Therefore, higher doses from very-early ages and a chronic administration should be necessary in order to protect from these symptoms.

Regarding cognitive abilities, an evident learning impairment was not detected in 3xTg-AD mice in the acquisition test at any studied age. At 7 and 9 months of age, 3xTg-AD mice needed to travel higher distances than NTg mice, which would suggest a mild impairment even though learning was evident. Nevertheless, an impairment in long-term memory was found at 5 months of age. In the probe test, 3xTg-AD mice showed a reduced number of entries and time spent in the platform zone and a reduced path efficiency to the platform. Moreover, 3xTg-AD mice travelled less distance than NTg mice in the platform zone and showed a random pattern in distance travelled in the different zones, compared with the platform-directed pattern shown by NTg mice. Interestingly an improvement in long-term memory over time could be detected specially in 3xTg-AD. Indeed, when they were 9 months old, differences in long-term memory were not found between 3xTg-AD and NTg mice. Overall, these results point to a beneficial effect of cognitive stimulation in ameliorating cognitive impairment in the 3xTg-AD mice model. Similarly, scFv-h3D6 affected long-term memory impairment detected at 5 months of age in the 3xTg-AD model. After scFv-h3D6 administration, differences between NTg and treated 3xTg-AD mice in number of entries, time spent and distance travelled in the platform zone, as well as in the path efficiency to the platform, were not observed, supporting a possible positive effect of anti-Aβ immunotherapy at this early stage of the disease. The effect of the treatment was not detected at 7 and 9 months of age, probably because training masked the effect of this low-doses treatment. Indeed, although a positive effect of scFv-h3D6 treatment at similar doses was observed in a longitudinal study followed by magnetic resonance imaging (MRI) [36], it is likely that the low dose used during both longitudinal studies could be increased to obtain more robust results, encouraging further studies with higher doses of this antibody fragment.

Moreover, it is important to note that our results indicate that longitudinal studies are not the best strategy to evaluate the effects of a chronic treatment, specifically when behavioral testing is needed. Interactions that cognitive stimulation have on learning and memory processes could mask, at least partially, the effects of the treatment. Thus, if the effects of a longitudinal treatment are to be assessed, it is more convenient to analyze the effects of the drug with different cohorts at different time-points. In summary, this study suggests that cognitive impairment early triggered by the pathology could be at least partially recovered from the first moment by a combination of immunotherapy and cognitive stimulation. Taking into account the multifactorial origin of AD, it is convenient develop combined therapies directed to different targets in order to find an effective therapy for this complex and devastating disease.

## Figures and Tables

**Table 1:** Corner test data. Values are expressed by means and SD. ^#^ indicates a low, but not significant, *p*-value 0.05 < *p* ≤ 0.1, * significant differences with *p* ≤ 0.05, ** *p* ≤ 0.01, *** *p* ≤ 0.001, and **** *p* ≤ 0.0001. ^A^ refers to NTg/- mice and ^B^ refers to 3xTg/- mice.

Corner Test	NTg	PBS-Treated 3xTg-AD	ScFv-h3D6-Treated 3xTg-AD
*Horizontal activity*			
Distance traveled (m)			
5-month-old	1.53 ± 0.14	1.90 ± 0.15 ^#A^	2.29 ± 0.20 **^A^
7-month-old	0.96 ± 0.06	0.51 ± 0.11 **^A^	0.56 ± 0.16 *^A^
9-month-old	0.50 ± 0.13	0.49 ± 0.16	0.49 ± 0.13
Corner visits (n)			
5-month-old	11.00 ± 0.68	10.80 ± 0.77	12.10 ± 1.08
7-month-old	6.50 ± 0.37	2.4 ± 0.69 ****^A^	3.10 ± 0.88 **^A^
9-month-old	5.20 ± 0.85	2.63 ± 0.58 *^A^	2.70 ± 0.65 *^A^
Corner latency (s)			
5-month-old	3.37 ± 0.63	1.76 ± 0.75	1.15 ± 0.41 **^A^
7-month-old	1.95 ± 0.43	10.67 ± 3.64 *^A^	10.67 ± 3.77 *^A^
9-month-old	1.94 ± 0.67	5.50 ± 2.43	4.88 ± 2.39
Time in the corner (s)			
5-month-old	10.56 ± 0.66	10.25 ± 1.12	9.26 ± 1.17
7-month-old	10.52 ± 1.08	5.99 ± 1.78 *^A^	4.70 ± 1.43 **^A^
9-month-old	9.08 ± 1.77	8.20 ± 2.93	7.71 ± 1.86
*Vertical activity*			
Rearings (n)			
5-month-old	4.15 ± 0.49	4.20 ± 0.51	3.70 ± 0.83
7-month-old	4.00 ± 0.45	2.67 ± 1.01	1.00 ± 0.33 ****^A^
9-month-old	2.80 ± 0.89	0.38 ± 0.24 *^A^	0.40 ± 0.16 *^A^
1st rearing latency (s)			
5-month-old	0.13 ± 0.02	0.13 ± 0.02	0.12 ±0.03
7-month-old	7.30 ± 1.24	18.00 ± 3.72 *^A^	23.00 ± 2.53 ****^A^
9-month-old	12.30 ± 3.18	25.88 ± 2.43 **^A^	25.40 ± 2.76 **^A^
*Other emotional behaviors*			
Immobility time (s)			
5-month-old	0.92 ± 0.50	0.65 ± 0.43	0.53 ± 0.35
7-month-old	6.06 ± 1.30	17.18 ± 2.00 ***^A^	18.54 ± 3.21 **^A^
9-month-old	12.73 ± 2.75	20.59 ± 2.50 *^A^	22.06 ± 1.46 **^A^
Defecations (boli)			
5-month-old	0.40 ± 0.22	0.30 ± 0.21	0.20 ± 0.13
7-month-old	1.00 ± 0.26	0.56 ± 0.28	0.50 ± 0.22
9-month-old	0.80 ± 0.20	0.25 ± 0.15 ^#A^	0.00 ± 0.00 ***^A,#B^

**Table 2:** Open-field test data. Values are expressed by means and SD. ^#^ indicates a low, but not significant, *p*-value 0.05 < *p* ≤ 0.1, * significant differences with *p* ≤ 0.05, ** *p* ≤ 0.01, *** *p* ≤ 0.001, and **** *p* ≤ 0.0001. ^A^ refers to NTg/- mice, ^B^ refers to the 3xTg/- mice and ^C^ refers to the 3xTg/WT mice.

Open-Field Test	NTg	PBS-Treated 3xTg-AD	ScFv-h3D6-Treated 3xTg-AD
*Horizontal activity*			
Total distance traveled (m)			
5-month-old	46.87 ± 3.54	19.36 ± 2.79 ****^A^	24.30 ± 2.88 ***^A^
7-month-old	22.49 ± 2.88	12.94 ± 3.75 ^#A^	9.23 ± 1.82 **^A^
9-month-old	17.54 ± 3.07	10.43 ± 2.05 *^A^	12.74 ± 2.75
Entries to the center (n)			
5-month-old	41.70 ± 5.32	23.50 ± 4.92 *^A^	26.60 ± 5.73 ^#A^
7-month-old	22.00 ± 2.77	13.20 ± 4.86	12.3 ± 3.81 ^#A^
9-month-old	15.6 ± 2.76	15.25 ± 4.64	14.10 ± 4.29
Distance in the center (m)			
5-month-old	6.61 ± 0.58	3.68 ± 0.73 **^A^	4.02 ± 0.88 *^A^
7-month-old	2.85 ± 0.45	2.10 ± 0.82	1.57 ± 0.52 ^#A^
9-month-old	1.93 ± 0.31	1.58 ± 0.45	1.91 ± 0.58
Distance in the periphery (m)			
5-month-old	40.26 ± 3.08	15.49 ± 2.20 ****^A^	20.29 ± 2.34 ****^A^
7-month-old	19.65 ± 2.52	10.83 ± 3.06 *^A^	7.66 ± 1.32 **^A^
9-month-old	15.61 ± 2.82	8.86 ± 1.61 *^A^	10.84 ± 2.22
Ratio center/periphery (dist)			
5-month-old	0.17 ± 0.01	0.24 ± 0.04	0.20 ± 0.03
7-month-old	0.15 ± 0.01	0.23 ± 0.05	0.17 ± 0.03
9-month-old	0.13 ± 0.02	0.15 ± 0.03	0.16 ± 0.03
Time in the center (s)			
5-month-old	76.75 ± 9.03	93.07 ± 23.87	75.20 ± 17.30
7-month-old	50.66 ± 7.40	159.5 ± 46.4 *^A^	52.46 ± 17.91 *^B^
9-month-old	38.99 ± 7.74	57.98 ± 25.95	68.30 ± 23.00
Time in the periphery (s)			
5-month-old	823.3 ± 9.04	723.2 ± 78.2	824.7 ± 17.3
7-month-old	849.3 ± 7.39	740.2 ± 46.34 *^A^	847.5 ± 17.9 *^B^
9-month-old	861.01 ± 7.73	841.98 ± 25.94	831.7 ± 23.0
Ratio center/periphery (time)			
5-month-old	0.09 ± 0.01	0.15 ± 0.04	0.10 ± 0.02
7-month-old	0.06 ± 0.01	0.27 ± 0.10 ^#A^	0.07 ± 0.02 ^#B^
9-month-old	0.05 ± 0.01	0.08 ± 0.04	0.09 ± 0.03
*Vertical activity*			
Rearings (n)			
5-month-old	73.20 ± 6.28	14.78 ± 3.22 ****^A^	14.50 ± 2.25 ****^A^
7-month-old	35.60 ± 7.26	6.60 ± 1.75 **^A^	3.80 ± 0.89 ***^A^
9-month-old	26.50 ± 6.36	3.57 ± 1.09 **^A^	5.10 ± 1.33 **^A^
1^st^ rearing latency (s)			
5-month-old	18.70 ± 3.10	64.89 ± 13.50 **^A^	54.40 ± 14.48 ***^A^
7-month-old	18.20 ± 4.36	360.9 ± 124.8 *^A^	243.3 ± 57.1 ****^A^
9-month-old	123.4 ± 87.7	269.1 ± 48.9	314.9 ± 105.1
*Other emotional behaviors*			
Immobility time (s)			
5-month-old	116.8 ± 38.44	304.95 ± 84.19 ^#A^	215.27 ± 88.67
7-month-old	229.41 ± 80.38	459.06 ± 107.27	310.07 ± 127.14
9-month-old	364.31 ± 108.38	285.86 ± 125.45	525.6 ± 115.84
Self-groomings (n)			
5-month-old	4.30 ± 0.34	3.33 ± 0.73	2.90 ± 1.06
7-month-old	3.90 ± 1.14	3.40 ± 0.75	2.40 ± 0.7
9-month-old	5.10 ± 0.74	3.50 ± 0.54	3.10 ± 0.38 *^A^
Self-groomings latency (s)			
5-month-old	148.4 ± 39.3	265.4 ± 31.8 *^A^	343.0 ± 98.8 ^#A^
7-month-old	276.5 ± 71.1	381.7 ± 77.9	402.3 ± 94.1
9-month-old	80.20 ± 12.76	177.4 ± 35.2 *^A^	201.9 ± 32.0 **^A^
Time on self-grooming (s)			
5-month-old	7.20 ± 0.98	5.24 ± 0.95	6.07 ± 2.31
7-month-old	7.35 ± 1.93	6.15 ± 1.63	5.10 ± 1.42
9-month-old	8.77 ± 1.42	7.46 ± 0.94	7.05 ± 0.77

## Abbreviations

3xTg-AD	triple-transgenic mouse model of AD
AD	Alzheimer’s disease
BPSD	behavioral and psychological symptoms of dementia
CT	corner test
MWM	Morris Water Maze
NTg	non-transgenic
OFT	open-field test
